# Defective and Epileptic Children

**Published:** 1898-08-20

**Authors:** 


### DEFECTIVE AND EPILEPTIC CHILDREN.

The report of the Departmental Committee on Defective and Epileptic Children which was published some time ago contains much that is of interest to medical practitioners. Although the facts elicited and recommendations made refer solely to children attending public elementary schools, yet they apply almost equally well to feeble-minded children of the middle and upper classes, and valuable hints as to the treatment and management of these most difficult cases may be obtained by a perusal of the report. Not only so, but should the proposals of the committee be followed by legislation there will then be created a large number of School Board medical officers, and the long disputed question of payment for medical certificates so far as this class of case is concerned will once for all be settled.

The report deals with feeble-minded children, who, whilst neither idiots or imbeciles, are yet incapable of being taught in ordinary schools by ordinary methods. There are already thirteen hundred such children under instruction in special classes, and the results generally are encouraging, it being found possible not only to impart a certain degree of manual skill, but also by the thirteenth or fourteenth year to bring them to about the same standard of elementary education as is reached by a normal child of eight or nine. For the great part such children exhibit certain bodily defects, irregular conformation, defective palate, tongue, teeth, lips, or ears, motor or sensory defects, or general bad nutrition. Speaking generally, one per cent. of children of the public elementary school class are feeble-minded, whilst, in addition to these, one or two per thousand are incapable from physical defects, such as lameness, fits, &c., from attending school. At the present time the authorities have no power to place feeble-minded children in special schools or classes without the parents' consent, nor, indeed, need they make any provision for such children. The committee recommend that in centres where the population exceeds 20,000, the provision of special classes and the drafting of feeble-minded children into such shall be compulsory. The method of selecting the children to be as follows: Up to the age of seven years no distinction will be made, children of all grades of mental development alike attending Board schools and infant schools. At the expiration of this time the head teachers must select such children as appear to them to be mentally deficient, and these are to be examined in the presence of parent, past and future teacher, and inspector, by an expert medical man, who will certify as to their fitness for ordinary school life, special school, or home for imbeciles or idiots. The parent, if aggrieved, may appeal to the Education Department. The special classes will be taught by trained teachers, and will not be of more than four and a half hours duration daily. At least six hours a week must be devoted to manual instruction for the younger children, knitting, modelling, colouring macramé, paper-folding, basket and mat-making. For the older children cane-seating, simple woodwork and joinery, basket, brush and matmaking for the boys, and needlework, cooking, and washing for the girls. Games, especially games with balls, breathing drill, and eye-movements, are to form part of the curriculum. The medical officer shall visit and examine the classes every year.

It is recommended also that conveyance or guides should be furnished to deficient children and to children physically unfit, and that where necessary children should be boarded out in the neighbourhood of the school rather than have long daily journeys. Institutions and "homes" are not, as a rule, favourable for such children.

Epileptic children, who number about one per thousand of the child population, are classified under two heads—(1) Mild cases of normal or of feeble intellect; and (2) severe cases.

Mild cases of epilepsy with no mental impairment are to be retained in the elementary schools, teachers being given special instructions with regard to treatment during fits, and also as to their general training. Mentally deficient children with mild epilepsy should be sent to classes for the feeble-minded, and all severe cases, having been medically certified as such, should be treated in small country sanatoria such as at present exist, or may at any future time be erected by School Board authorities. In an appendix Dr. Shuttleworth, a member of the committee, gives examples of physical defects found in feeble-minded children, together with suggestions for the modifications in education which such defects require.

From what we have said it is evident that if the recommendations of the Committee be embodied in legislation, medical men will play a much more prominent part than formerly in the educational methods of the country.

